# Clinical outcomes of arthroscopic and navigation-assisted two tunnel technique for coracoclavicular ligament augmentation of acute acromioclavicular joint dislocations

**DOI:** 10.1186/s12891-021-04406-2

**Published:** 2021-06-09

**Authors:** Jan Theopold, Ralf Henkelmann, Claus Zhang, Tobias Schöbel, Georg Osterhoff, Pierre Hepp

**Affiliations:** Division of Arthroscopy, Joint Surgery and Sport Injuries, Department of Orthopedics, Trauma, and Plastic Surgery, University of Leipzig, Liebigstrasse 20, 04103 Leipzig, Germany

**Keywords:** Acromioclavicular joint, Clavicle, Shoulder, Joint stability, Articular ligaments

## Abstract

**Background:**

The purpose of this study was to present a navigated image-free augmentation technique for the acromioclavicular joint (ACJ) and coracoclavicular (CC) ligaments and to report the clinical and radiological outcomes.

**Methods:**

From 2013 to 2018, 35 eligible patients were treated with our navigated image-free ACJ- and CC-augmentation technique. The average follow-up was 3 years. Follow-up evaluations included the Constant-Murley Score, subjective shoulder value, Taft score, and the acromioclavicular joint instability (ACJI) score. The patients’ quality of life was assessed using the EuroQol-5D (EQ-5D) questionnaire. In addition, in accordance with the instability criteria, radiographs were evaluated before surgery, after surgery, and during follow-up.

**Results:**

Overall, 25 patients (71%) suffered an acute type V disruption, 5 (14%) had a type IV disruption, and 5 (14%) had an acute Rockwood type IIIb injury. The mean Constant-Murley Score was 90 (range: 56–100; *p* = 0.53) on the injured side, and the mean subjective shoulder value was 92% (range: 80–100%). The mean Taft and ACJI scores were 10 (range: 4–12) and 86 (range: 34–100), respectively and the mean EQ-5D was 86 (range: 2–100). The mean CC difference of the injured side was 4 mm (range: 1.9–9.1 mm) at follow-up, which was not significantly different than that of the healthy side (*p* = 0.06). No fractures in the area of the clavicle or the coracoid were reported.

**Conclusions:**

The arthroscopic- and navigation-assisted treatment of high-grade ACJ injuries in an anatomical double-tunnel configuration yields similar clinical and radiological outcomes as the conventional technique using an aiming device. Precise positioning of the navigation system prevents multiple drillings, which avoids fractures.

**Supplementary Information:**

The online version contains supplementary material available at 10.1186/s12891-021-04406-2.

## Background

Shoulder injuries account for 7% of all sports injuries [1]. Of these, 9% are injuries to the acromioclavicular joint (ACJ) [2, 3]. Nearly half of the patients with ACJ injuries (43.5%) are 20–30-year-old males [3]. The mechanism of injury is typically direct trauma caused by a fall or a blow to the arm in the adducted position [3]. Contact sports, such as rugby or American football, are high-risk activities for ACJ injuries [4].

There are a variety of treatment options for high-grade ACJ injuries [3, 5]. Common surgical approaches for reconstructing the ACJ include repairing the acromioclavicular (AC) complex [6] and reconstructing the coracoclavicular (CC) ligaments [7]. A combination of both techniques is often recommended [3, 8, 9]. The use of arthroscopic techniques in the surgical treatment of ACJ injuries is increasing, including the augmentation of the CC ligaments with suture based reconstruction in a double-tunnel (DT) technique, [10–12] which yields favorable clinical outcomes [11, 13].

A disadvantage of most CC augmentation techniques is that one or more arthroscopically-assisted drillings of the coracoid are required. When multiple attempts are needed to find the optimal drill hole position, fractures of the coracoid can occur, [14–17] resulting in construct failure and poor outcomes [14–16]. Navigation-assisted techniques can be used to avoid multiple drilling attempts [18–21]. The aim of this study was to present a navigation-assisted augmentation technique for the ACJ and CC ligaments and to report the clinical and radiological outcomes of this technique.

## Methods

### Patients

Thirty-five consecutive patients with acute ACJ dislocations who were treated via arthroscopic and navigation-assisted DT procedures from 2013 to 2018 were included in this retrospective study [22, 23]. All interventions were performed by two experienced joint surgeons using the same suture system and our navigation technique. All operations where done within a period of 2 weeks after the injury. This study was approved by the local institutional ethics committee and follows the principles of the Declaration of Helsinki. Informed written consent was obtained from each patient.

### Surgical technique

A standard diagnostic arthroscopy of the shoulder joint was performed under general anesthesia with the patient in a beach chair position (see Additional file 1). In cases of concomitant glenohumeral injuries, these injuries were treated first. Following arthroscopy, an anterior-inferior working portal was created just above the subscapularis tendon and a lateral viewing portal was created using the outside-in technique. Next, the subcoracoid space and the base of the coracoid were prepared using a radiofrequency ablation device inserted through the anterior-inferior portal.

A 5-cm sagittal saber incision was made across the clavicle approximately 1.5 cm medial to the ACJ. The deltotrapezial fascia was identified, and a T-shaped incision of the fascia was made over the ACJ and lateral clavicle. The ACJ was freed from the wrapped soft tissue of the AC capsule and AC ligaments. If severely damaged, the articular disc was excised. After an open reduction of the ACJ, AC transfixation was performed using a K-wire. The correct reduction of the joint was evaluated using an image intensifier.

### Reconstruction of the ligaments

Two holes were drilled in the clavicle for the reconstruction of the CC ligament. The first followed the course of the conoid ligament with a 2.4-mm K-wire from the clavicle to the coracoid, 5 mm medial to the isometric point of the clavicle, as defined by Rios [24]. The target zone was the posterior side of the coracoid base, 5 mm lateral to the medial boundary. The second hole followed the course of the trapezoid ligament, beginning 5 mm lateral to the isometric point of the clavicle. The target zone for the second coracoid tunnel was 10 mm anterior to the conoidal tunnel and 5 mm medial to the medial edge of the coracoid, with the intention of leaving a bony bridge of at least 10 mm between the tunnels [12, 25].

An established optoelectronic system with a corresponding software module was used for navigation (Trauma 2D 3.1 software, produced by Brainlab AG, Munich, Germany). Reflective markers were attached to the pointer to determine its position and to the drill sleeve to navigate the drilling direction. A 3D camera enabled real-time tracking of the drill sleeve in relation to the pointer. The movement of the two instruments was controlled in three projections (front, top, and overview), which were displayed on a touchscreen. A virtual red line marked the tips of both instruments and showed the target trajectory. The corresponding drilling direction of the sleeve was then compared with the red line to reach the corresponding target point. An autopilot was used to orient the navigated instruments, similar to its recent application in trauma software [18, 20].

After the instruments had been calibrated, the tip of the pointer was positioned at the subcoracoid target area through the anterior-inferior portal under arthroscopic control. The inserted K-wires were drilled over with a cannulated drill (4.0 mm). Two suture cerclage systems (TightRope, Arthrex, Inc., Naples, Florida, USA) were introduced into the CC tunnel with an insertion aid (Application Sleeve & Pusher, Arthrex, Inc., USA) until the oval buttons could be anchored under the coracoid arch under arthroscopic control. The thread systems were tensioned by alternating tension between the two implants. Finally, the threads were tied proximally.

The detached deltoid and trapezius fascia were anatomically reattached to the lateral clavicle using transosseous sutures (#2 FiberWire, Arthrex, Inc., Naples, Florida, USA), and the complete closure of the fascia was accomplished using a fascia suture (Vicryl size 1, Ethicon, Norderstedt, Germany). The initial T-shaped incision of the AC capsule was closed using a 3.5-mm suture anchor (Arthrex, Naples, Florida, USA) on the lateral clavicle. Finally, the temporary ACJ K-wire transfixation was removed using the image intensifier. The upper incision was sutured in layers, and the arthroscopic portals were closed using conventional methods.

### Postoperative care

During the immediate postoperative period, the shoulder was immobilized via an internal rotation sling (shoulder immobilization support, Medi GmbH & Co. KG, Bayreuth, Germany). Patients were permitted to perform passive movement exercises up to a flexion and abduction of 45° for the first 3 weeks postoperatively and up to 90° during the subsequent 3 weeks. Active movement exercises were permitted beginning in postoperative week 7. Patients were advised to avoid exercises that stressed the ACJ, such as grasping, pushing, and pulling during that time.

### Clinical and radiological evaluation

**Fig. 1:**
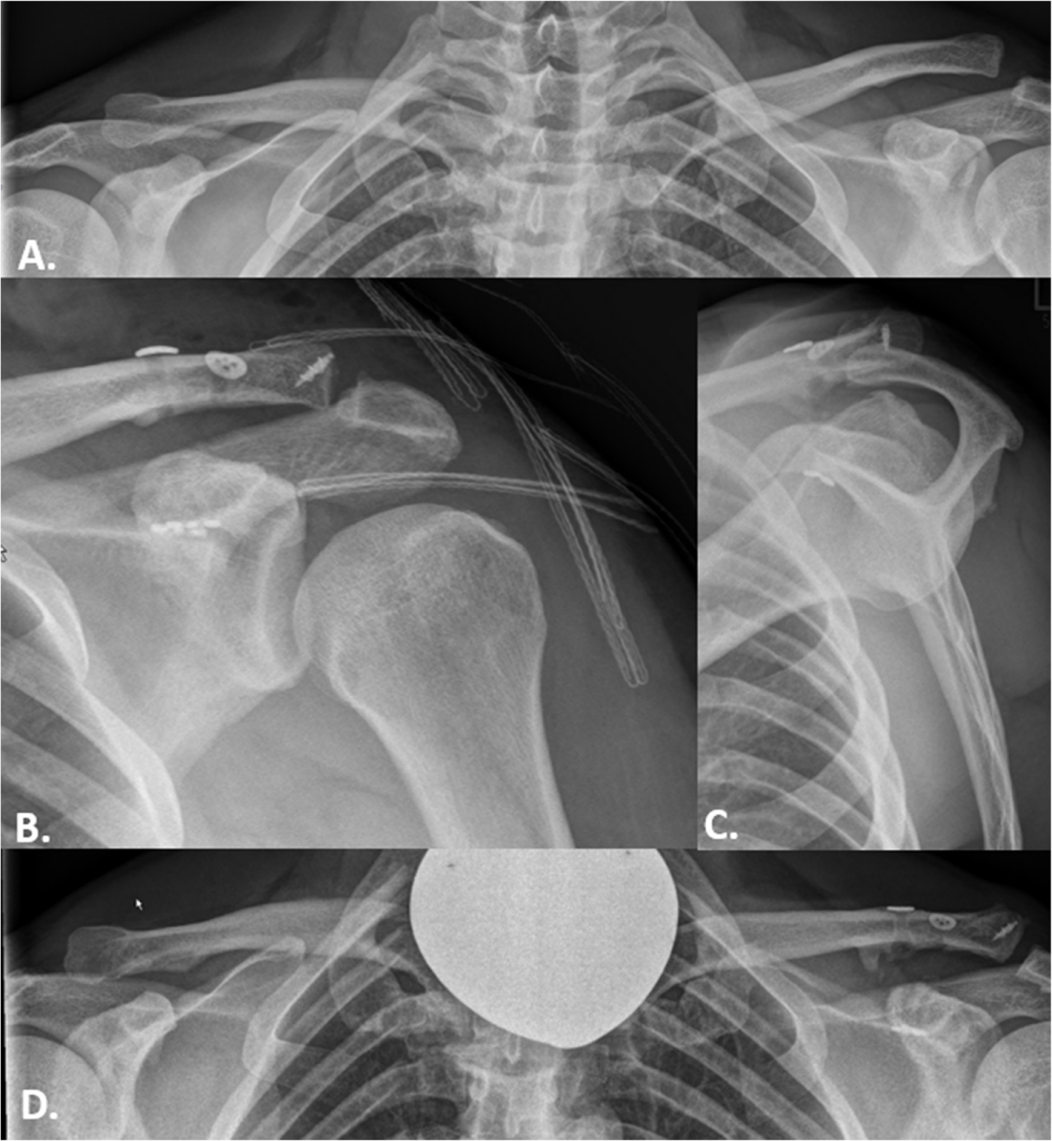
Case presentation. Left: A 42-year-old patient with an acromioclavicular joint injury due to a contact sport. **A**. A panoramic radiograph shows a Rockwood type IIIb injury. **B** and **C**. Radiographs obtained in the immediate postoperative period show the correct reduction of the ACJ (B. anterior-posterior and C. Alexander view). **D**. A radiograph obtained 67 months postoperatively shows good reduction with subtle ossification in the ligament area. The implants are without secondary dislocation (panorama view)

The clinical evaluation (Fig. 1) consisted of a complete physical examination of both shoulders and several shoulder function evaluations, including the Constant-Murley score (CMS), subjective shoulder value (SSV), Taft score (TF), and acromioclavicular joint instability (ACJI) score [11, 26–28]. The TF was described by Taft et al. 1987 [26]. It grades results after conservative and surgical treatment of AC joint dislocations. Subcategories are “subjective”(=pain; 4 points), “objective” (=range of motion and strength; 4 points) and “radiologic” (4 points). So, the maximum score is 12 points. Points can be subtracted for different symtoms: tenderness to palpation of the AC-joint, bad cosmetic results, or crepitation. Force measurements were performed on both shoulders with the aid of an isometric dynamometer (Isobex TM dynamometer, MDS Medical Device Solutions AG, Burgdorf, Switzerland). Quality of life was assessed using the EuroQol-5D (EQ-5D) questionnaire [29, 30].

Panoramic images were obtained with an axial load of 10 kg (Fig. 1). The CC distance was measured as the distance between the upper edge of the coracoid and the lower cortex of the clavicle. The measurement was performed on both shoulders preoperatively, during the immediate postoperative period, and during follow-up.

Horizontal stability was assessed via bilateral Alexander views on radiographs obtained during follow-up (Fig. 1C) [31–34]. Patients were classified into three groups according to the ACJI score: stable ACJ, partially stable ACJ, and unstable ACJ [35].

### Statistical analysis

Baseline characteristics are presented as mean (standard deviation) for continuous data and frequency (percent) for categorical data. The CMS was calculated for individual parameters using Microsoft Excel. The indices of the EQ-5D were calculated using the Crosswalk Index Value Calculator tool developed for Germany (EuroQol Research Foundation, Rotterdam, Netherlands). A Mann-Whitney U test was used to detect differences between the means of continuous data, and the chi-square test or Fisher’s exact test was used to identify differences between categorical data. Statistical calculations were performed using IBM-SPSS version 24 software (IBM, New York, USA). The level of significance was set at *p* < 0.05.

## Results

Thirty-five patients (30 males and 5 females) with a mean age of 40 years (range: 23–57 years) were included in this study. The mean follow-up period was 37.8 months (range: 14.7–67 months). Twenty-five patients (71%) suffered an acute Rockwood type V disruption, 5 (14%) had a Rockwood type IV disruption, and 5 (14%) had an acute Rockwood type IIIb injury [36, 37]. The patients’ epidemiological data are shown in Table 1.

**Table 1:** Patient epidemiological data

	Mean	Range	Standard deviation
Age, years	40.2	23–57	9.64
Follow-up, months	37.8	14.7–67.1	17.50
	n	Percent	
Sex			
Male	30	85.7	
Female	5	14.3	
Rockwood type injury			
III	5	14.3	
IV	5	14.3	
V	25	71.4	
Injured side			
Right	21	60.0	
Left	14	40.0	
Handedness			
Right	31	88.6	
Left	2	5.7	
Ambidextrous	2	5.7	
Cause of injury			
Bicycle fall	25	71.4	
Handball	2	5.7	
Ski/snowboard	3	8.6	
Fall	5	14.3	
Complaints of shoulder injury			
Yes	4	10.4	
No	31	89.6	
Return to work			
Yes	34	97.1	
No	1	2.9	
Incapacity to work			
Yes	1	2.9	
No	33	94.3	
Partial	1	2.9	
Change of work duties			
Yes	1	2.9	
No	34	97.1	

Incision to suture time alone for all surgeries averaged 75.2 ± 17.7 min (46–130 min). In patients with additive injuries to the glenohumeral joint (e.g., SLAP lesions, pully injuries), the incision-suture time was 85.6 ± 20 min (50–130 min), and in patients without a corresponding additive injury, it was 68.4 ± 11.2 min (46–97 min).

### Clinical outcomes

**Fig. 2:**
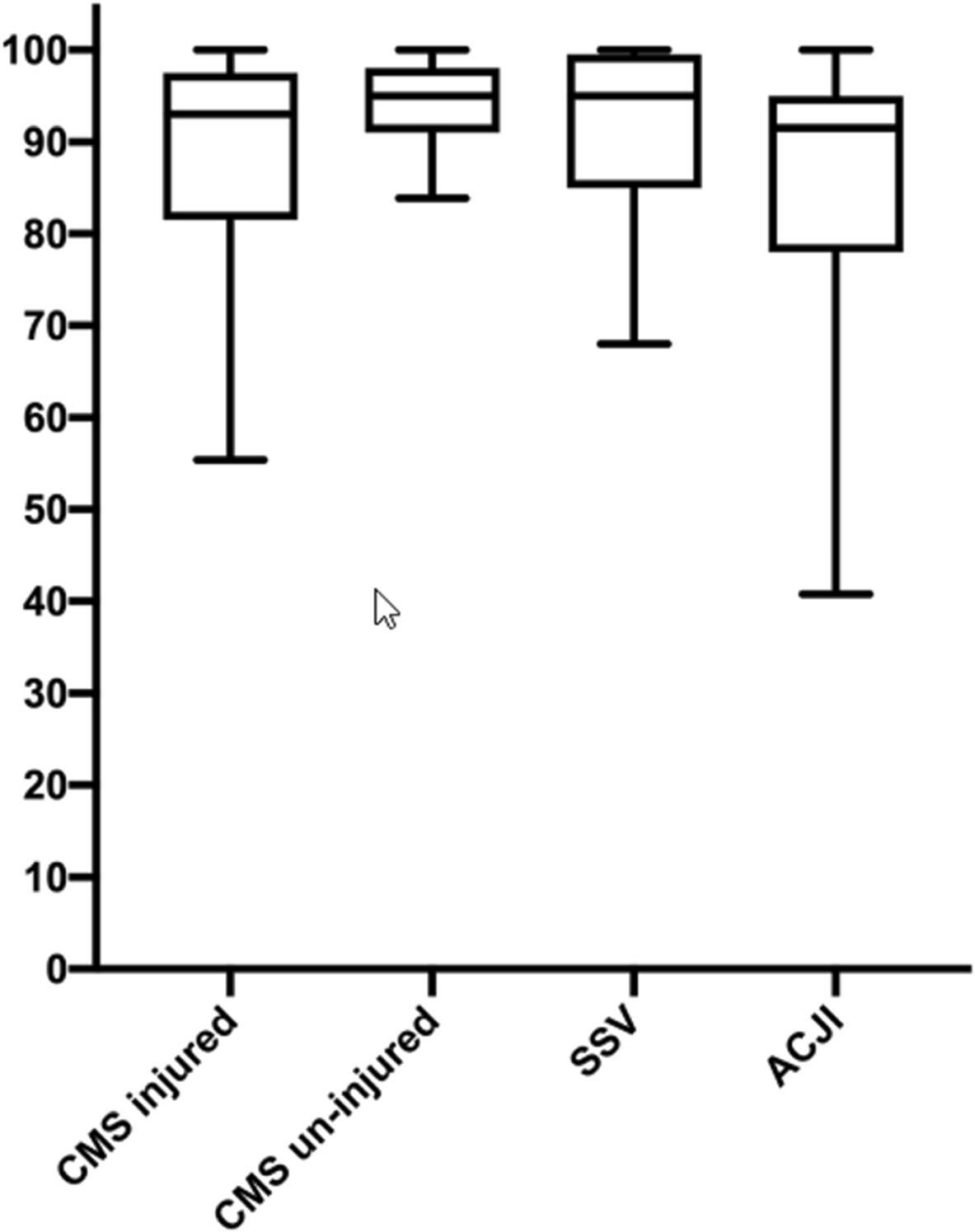
Clinical outcomes. The Constant-Murley Score (CMS), subjective shoulder value (SSV), and acromioclavicular joint instability (ACJI) score were calculated for each patient. Averages of these scores are shown in a boxplot. The CMS of the injured shoulder was not significantly different than that of the un-injured side (*p* = 0.53)

The mean CMS on the injured side was 90 (range: 56–100) and on the contralateral side was 95 (range: 89–100) (p = 0.53). The mean SSV was 92% (range: 80–100). The mean TF was 10 (range: 4–12). The mean ACJI score was 86 (range: 34–100) (Fig. 2). The mean EQ-5D was 86 (range: 2–100) (Table 2).

**Table 2:** Clinical score outcomes

		Mean	Range	Standard deviation
EuroQOL-5D Questionnaire				
Pain		16.0	5–20	5.11
Everyday activity		9.3	5–10	1.78
Cosmetics		9.0	0–10	2.66
Function		20.4	0–25	6.34
Radiological outcome		31.4	9–35	6.56
Total		85.7	34–100	17.29
Constant-Murley Score	Injured	89.7	56–100	10.19
	Uninjured	95.0	89–100	3.96
Taft Score		9.8	4–12	1.85
Subjective Shoulder Value		92.43	80–100	6.87

### Radiological outcomes

**Fig. 3:**
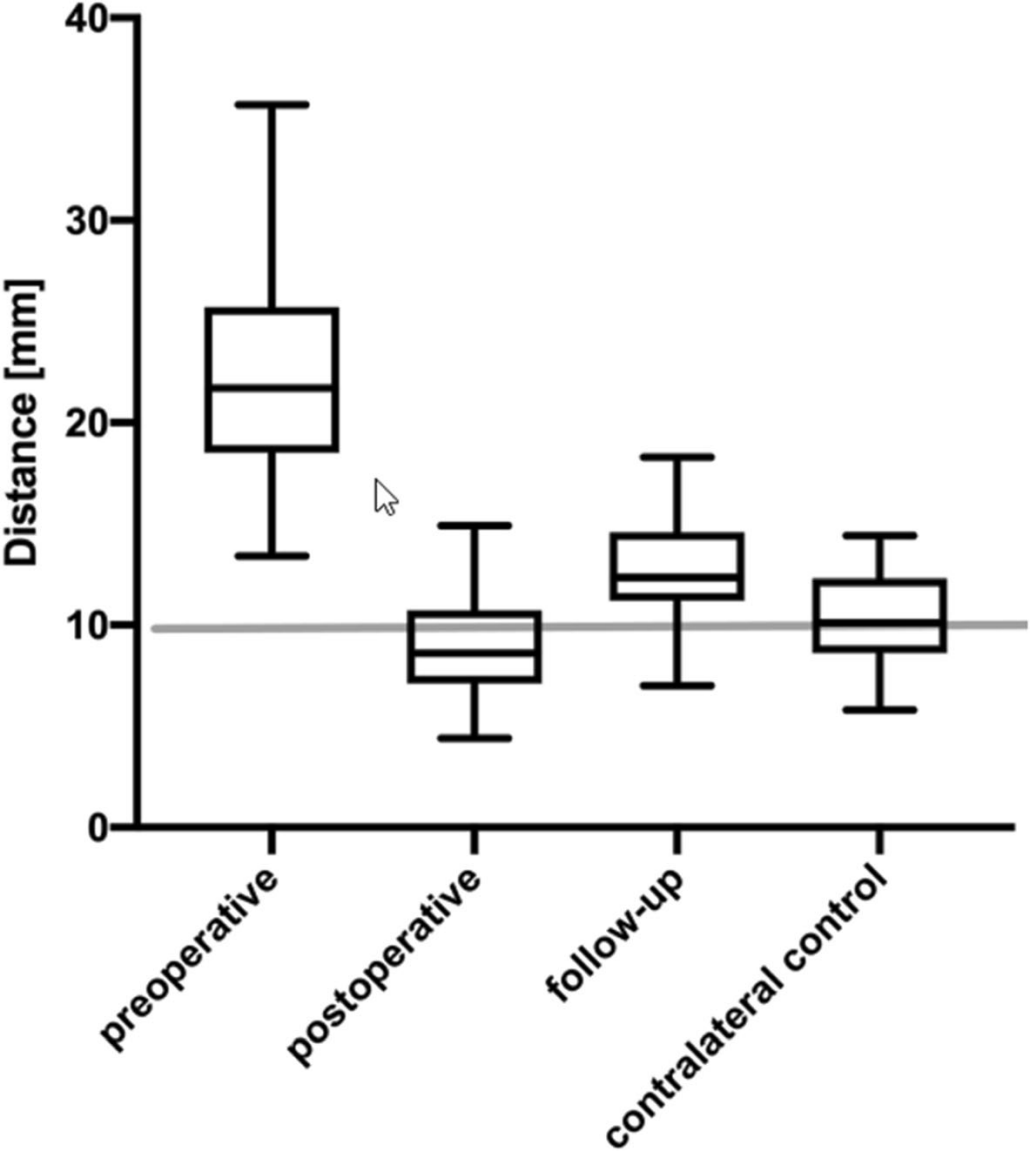
Coracoclavicular distance. The coracoclavicular (CC) distance was measured preoperatively, in the immediate postoperative period, and during follow-up in the injured shoulder. The final CC distance was not significantly different from that in the contralateral shoulder (*p* = 0.06)

The mean preoperative CC distance of the affected shoulder was 22.6 mm (range: 13.4–35.7 mm), and the mean postoperative CC distance was 8.8 mm (range: 4.4–14.9 mm). The mean CC distance of the healthy shoulder was 10.3 mm (range: 5.8–14.4 mm; *p* = 0.8). The mean CC difference during the follow-up period was 4 mm (range: 1.9–9.1 mm), which was not significantly different than that of the healthy shoulder (*p* = 0.06) (Fig. 3).

The Alexander view of postoperative radiographs revealed 1 patient (3.1%) with complete instability of the ACJ and 11 patients (34.4%) with partial instability. Of the patients with Rockwood type V injuries, none had complete instability and 10 (40%) had partial instability.

### Complications

#### Intraoperative complications did not occur

Overall, no fractures in the area of the clavicle or in the area of the coracoid were found on any radiological examination.

In four patients, the material was removed due to thread irritation after 9, 12, 18, and 36 months. No postoperative infections or wound healing disorders occurred.

## Discussion

The most important findings of the present study were that no fractures occurred in the area of the clavicle or coracoid and no secondary dislocations of the distal buttons of the coracoid occurred. The arthroscopic- and navigation-assisted treatment of high-grade ACJ injuries in an anatomical double-tunnel configuration yielding similar clinical and radiological outcomes as the conventional technique, suggesting the effectiveness of the navigation-assisted technique used in this study for ACJ and CC ligament reconstruction.

Conventional CC augmentation techniques have an increased risk of fracturing the clavicle or coracoid due to multiple drilling attempts. Previous studies have reported that two holes should not be attempted, especially on the coracoid [14–17]. Various biomechanical studies have shown that optimal vertical and horizontal stability can be achieved via the most anatomical reconstruction of the CC ligament possible [10–12, 23]. As anatomical reconstruction of the drill channels is not possible using a rigid aiming device [5], other treatment options have been developed, including the minimally invasive additive ACJ cerclage [38–40]. Intraoperative navigation enables anatomical placement as well as a significant reduction in the number of incorrect drill holes [19–21, 41, 42].

Several studies report the use of an additive treatment of the AC ligaments to improve stability [38, 39, 43–45]. Our navigation-assisted technique includes the anatomical placement of the drill channels and direct suturing of the fascia and the AC ligaments via a minimally invasive approach for CC augmentation. Direct suturing of the fascia, AC ligaments, and AC capsule is necessary, as the indirect treatment of the complex disruptions of the affected structures is not sufficient [46]. The direct AC capsule suture may account for the low number of recurrent horizontal instabilities found in this study (*n* = 10, 40%). Hann et al. reported that 46.9% of patients with Rockwood type V injuries experienced instability after having undergone the indirect AC augmentation technique described in that study [35]. Biomechanical studies suggest that exact anatomical CC reconstruction has a positive impact on horizontal instability [23, 45, 47].

The CMS and SSV in this study are consistent with those of other studies [10, 11, 13, 35]. The T*F* is also comparable to previously reported studies [13, 44]. The validity of the EQ-5D for traumatic ACJ injuries has not yet been established. However, it has been reported to be comparable to the DASH (Disabilities of the Arm, Shoulder and Hand Score) and CMS [29, 48]. The EQ-5D scores in this study indicate good to very good overall health compared to published data of a general populations [30, 49, 50].

The ACJI scores reported in this study are comparable to those of Hann et al. [11, 13, 35], and higher than those of Kraus et al. [13]. These results may be due to the more favorable horizontal stability results obtained in this study.

The radiological assessment conducted during the postoperative period revealed a good reduction with minimal overcorrection and a secondary loss of reduction in the follow-up examinations. This loss of reduction is also described in other studies [11, 13], though the secondary dislocation rate is lower in this study than in a previous multicenter study [14].

Compared to other reconstruction methods like hook plates, our results are similar. Values around 90 points are also achieved with this method in the Constant score [51–53]. Hence, osteolysis in the acromion due to the hook of the plate and an increased visual analog scale for pain seem to occur more frequently [53–56].

### Limitations

This study is not without limitations. The follow-up duration of 31 months is relatively short and no conclusions regarding the long-term effects or the development of osteoarthritis can be made. Unfortunately, a comparison group was not possible in the study setup. For this reason only the comparison with the literature was carried out. Additionally, a direct measurement of the drill holes via computed tomography to verify their position was not performed due to the radiation exposure and the young age of the patients. A complete clinical and radiological examination was performed at the final time point shown. Interim examinations at 6 and 12 months were performed irregularly and were not included in the analysis. The influence of open reconstruction of the AC ligaments, capsule, and fascia cannot be reliably differentiated from the anatomical configuration. At minimum, this study shows comparable or better outcomes for horizontal instability (based on the Alexander views and ACJI scores) compared to other clinical studies on the reconstruction of the ACJ [11, 13, 14].

## Conclusion

The arthroscopic and navigation-assisted treatment of high-grade ACJ injuries in an anatomical DT configuration with direct suturing of the AC ligaments, capsule, and fascia is as effective as conventional methods using an aiming device in terms of clinical and radiological outcomes. Precise positioning of the navigation system prevents multiple drillings, which avoids fractures.

## Supplementary Information

**Additional file 1.**

## Data Availability

The datasets used and/or analyzed during the current study are available from the corresponding author on reasonable request.

## Abbreviations

AC: Acromioclavicular
CC: Coracoclavicular
ACJ: Acromioclavicular joint
DT: Double-tunnel
CMS: Constant-Murley score
SSV: Subjective shoulder value
TF: Taft score
ACJI: Acromioclavicular joint instability score
EQ 5 D: EuroQol-5D
DASH: Disabilities of the Arm, Shoulder and Hand Score
