# Supplementary material for: Clinical outcomes of arthroscopic and navigation-assisted two tunnel technique for coracoclavicular ligament augmentation of acute acromioclavicular joint dislocations
Source: BMC Musculoskelet Disord. 2021 Jun 9;22:528. doi: 10.1186/s12891-021-04406-2 (PMC8191052; doi:10.1186/s12891-021-04406-2)
Supplement: Supplementary file 1 — Additional file 1. [file 12891_2021_4406_MOESM1_ESM.docx]

Clinical outcomes of arthroscopic and navigation-assisted two tunnel technique for coracoclavicular ligament augmentation of acute acromioclavicular joint dislocations

Jan Theopold^¶*1^, Ralf Henkelmann^¶1^, Claus Zhang^1^, Tobias Schöbel^1^, Georg Osterhoff^1^ and Pierre Hepp^1^

1.) Department of Orthopedics, Trauma, and Plastic Surgery, University of Leipzig

Liebigstrasse 20, 04103 Leipzig, Germany

***Corresponding author**

Jan Theopold, PD Dr. med. habil., M.D.:

University of Leipzig, Department of Orthopedics, Trauma and Plastic Surgery, Division of Arthroscopy, Joint Surgery and Sport Injuries

Liebigstrasse 20, 04103 Leipzig, Germany

Tel: +49 341 9717386

Fax: +49 341 9723209

Email: jan.theopold@medizin.uni-leipzig.de

ORCID ID: orcid.org/0000-0003-2884-8030

^¶^ These authors contributed equally to this publication.

**Supplementary files**

**Title:** ACJ Rupture Rockwood V, Arthroscopic navigated Reconstruction.

Pierre Hepp, Jan Theopold, Ralf Henkelmann, Tobias Schöbel, Peter Melcher, Claus Zhang.

The video shows the surgical technique of the arthroscopic stabilization of an acute traumatic acromioclavicular joint separation in a 36-year-old male patient. The procedure is supported by navigation to avoid multiple drillings, especially in the coracoid area.
